# Emotion Science in the Twenty-First Century. Time, Sex, and Behavior in Emotion Science: Over and Above

**DOI:** 10.3389/fpsyg.2017.01211

**Published:** 2017-07-21

**Authors:** Marina A. Pavlova

**Affiliations:** Department of Psychiatry and Psychotherapy and Department of Biomedical Magnetic Resonance, Medical School, Eberhard Karls University of Tübingen Tübingen, Germany

**Keywords:** emotion science, brain imaging, gender/sex differences, brain networks, behavior, time course

With the advent of sophisticated tools and techniques over the past three decades, brain imaging has energized the rapidly developing field of Social Neuroscience, and has sparked a wide range of research in Emotion Science. Since its launch in 2010, *Frontiers in Emotion Science* has published more than 400 articles, many of them utilizing brain imaging tools. However, the question arises: Are we on the way to real progress? It is reasonable, and healthy, to reflect on how brain imaging has helped to elucidate our understanding of socio-emotional functioning, and how its contributions can be enriched and improved in the future. As a neuroscientist who has conducted many brain imaging studies, I wrestle with several doubts and concerns. In this piece of work, I will discuss the critical importance of *behavior, time*, and *sex/gender* not only for Emotion Science, but also “over and above” this specific area of investigation. I will focus on the ways in which these key constructs represent a grand (and very exciting) challenge for neuroscience. I elaborate on three of these here.

## Yes, look at behavior!

Even for the most enthusiastic colleagues, it is becoming more and more evident that functional brain imaging is not at all a “magic tool” that routinely offers desirable solutions for our understanding of the social brain and behavior, and, in particular, real-world affectively-motivated behavior. Decisive steps in understanding and explaining the multitude of subtle and sublime, emotionally meaningful, daily-life behaviors can be made only by carefully studying *behavior itself* (see also Beatrice de Gelder's analysis: “*Behavior, what else*?”; de Gelder, 2017). Of course, this does not mean that brain imaging does not provide us with valuable information about typical and aberrant affective processing. We might even think of brain imaging as providing a quantitative, reliable assay of the “behavior” of the nervous system. Yet (though it appears quite obvious, but hopefully not too banal), a relationship between behavioral performance during brain imaging and the patterns of change in brain activity should be in focus of investigation. Unfortunately, this is not always the case. This relationship is far from simple even in healthy adults, free from the rich complexities of developmental change or psychopathology: sometimes there is a strong positive correlation between behavioral measures of performance and brain activation suggesting that a higher performance level is accompanied by greater brain engagement (e.g., Grossman et al., 2004). In other cases, however, this correlation is negative (e.g., Raichle et al., 1994). This means that in those cases higher performance is accompanied by lower signal strength in the responsive brain regions. Why does this happen? Does greater functional brain activation points to greater task-related engagement of brain areas? If so, why is a more robust brain response needed for an easier or less demanding task, where participants exhibit ceiling levels of performance? These issues become even more challenging to understand when comparing performance and brain activations in individuals with atypical social cognition (e.g., in the presence of neurodevelopmental disorders such as autism and schizophrenia) to those with typical developmental pathways.

The question of how to investigate behavior, particularly, in relation to Emotion Science, is even more complicated. There is an understandable push to rapidly move to real-life situations and to leverage the possibilities afforded by virtual reality. However, by watching affective behavior of people in natural environment or “in the wild,” one confronts the ethical issues of how far and how deep to go when privacy might be violated? At the same time, simple awareness of the fact that “we-are-under-observation” leads to substantial alterations in behavior. “People-under-observation” may exhibit rather strong experimenter expectancy: they may wish to please the experimenter or behave in a socially desirable way suppressing either negative or positive (depending on cultural norms) emotional expressions. These are all well-known “ancient” problems of psychological research. But new tools reintroduce old problems and raise new ones as well: although virtual environments offer promising tools for studying more realistic affective behavior, the “question of belief” again comes into a play. Participants are aware that this reality is only virtual and this awareness potentially affects their behavior and emotional experience.

On a more positive note, we are clearly on the path to progress in studying dynamic instead of static (such as photographs, still images, depictions) affective faces and bodies (de Gelder, 2009; Kret and De Gelder, 2012; Pavlova, 2012), and (how delightful!) we are “standing up for the body” (de Gelder et al., 2010) and exploring body language reading in typical (Atkinson et al., 2004, 2012; Chouchourelou et al., 2006; Ikeda and Watanabe, 2009; Alaerts et al., 2011; Sokolov et al., 2011; Krüger et al., 2013; Actis-Grosso et al., 2015; de Gelder et al., 2015; for review, see Pavlova, 2017) and atypical development (e.g., Nackaerts et al., 2012; Strauss et al., 2015; Van den Stock et al., 2015; Vaskinn et al., 2016; Blain et al., 2017).

## Time matters

To date, brain imaging faces with a set of issues that must be addressed. Most prior work was restricted to localization of brain areas, and often referred to (in an unfortunately derisory fashion) as “blobology.” The “blobs” of activation are now recognized or believed to represent not only isolated icebergs of brain activation, but also hubs (i.e., key nodes for information processing as well as receptacles for connections to other brain regions) of the networks or neural circuits underlying complex cognitive and affective processes. Topography of these areas as well as the strength of activation vary meaningfully even with subtle alterations in the visual input, task demands, and participants' characteristics such as gender (Anderson et al., 2013; Pavlova et al., 2015), age (Ross et al., 2014), and socio-economic status (Muscatell et al., 2012). Recent work is beginning to focus on interactions between brain regions making up the social brain. One of the most desirable appeals for future work concerns re-definition of the concept of functional brain network both theoretically and experimentally (e.g., special issue in *Frontiers Systems Neuroscience*; *New concepts in brain networks*, Eds. Turner and Lohmann, 2012; Pessoa, 2014; Lohmann et al., 2016). Neuronal communication of the entire social brain should be a target of study in typical and atypical development. This goal demands a combination of advanced multimodal brain imaging tools allowing for assessment of temporal and spatial dynamics [such as magnetencephalography (MEG) and electroencephalography (EEG) along with (ultra) high field functional magnetic resonance imaging (fMRI) providing for high sensitivity and spatial resolution], and thereby, unveiling neural communication in real time. *Time* is a key to understanding the organization of functional brain networks, since brain topography alone do not allow us to understand neural communication as well as feed-forward and feed-back connections in the brain. Only the accurate measurement of temporal dynamics, in the context of spatial localization, provides the precise information about the formation of specialized functional brain networks, i.e., about brain areas and large-scale brain ensembles playing in unison at different time intervals.

**Figure 1:**
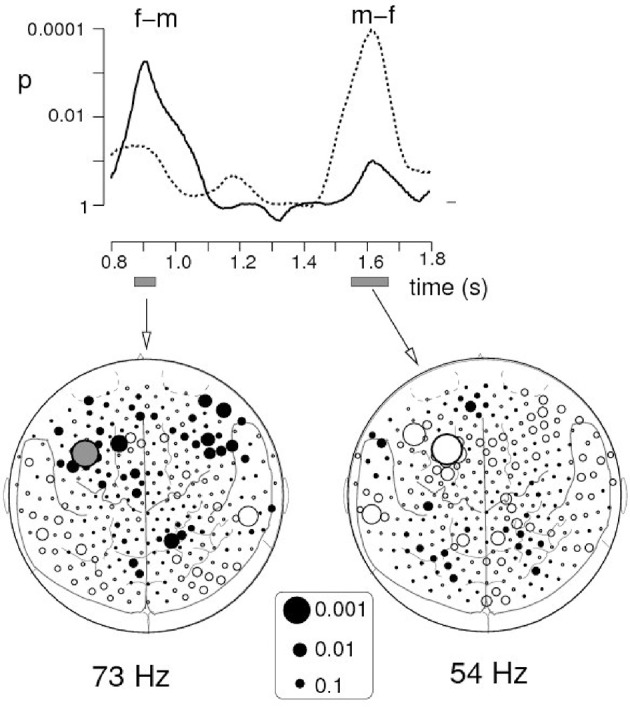
Sex effects on induced oscillatory gamma MEG activity. The graph on the top represents the outcome of *t*-test comparisons between females and males, i.e., the time course of the p values of the differences in the spectral amplitude in the filtered frequency bands. The solid curve shows the time course for differences in spectral amplitude between Heider-and-Simmel (HS) and control displays in females as compared with males at one of the sensors in the left prefrontal area (filled gray circle on the left map), whereas the dotted curve represents differences in males as compared with females at the sensor of similar topography (largest open circle on the right map). The culmination point of visual social interaction occurred at 1.3 s after stimulus onset. The left and right maps depict the topography of spectral amplitude differences in the 73-Hz and the 54-Hz ranges, respectively. Each circle represents one of the 275 MEG sensors projected onto a 2-dimensional cortical surface map with major anatomical landmarks (dorsal view, nose up). The size of the circle reflects the statistical strength of sex differences in induced oscillatory gamma activity. From Pavlova et al. (2010). © 2009 Elsevier Inc. with permission of Elsevier.

The other equally important reason for emphasizing *time* is that differences (sex-, age-, and psychopathology-related) in the *topography* of brain responses are often subtle or negligible: topographically similar brain areas or clusters of activation may be engaged in different groups of participants. What really matters is time! Temporal dynamics often helps to differentiate individuals and groups in spatial context. Let me give just one example from our own work in which we studied sex differences in the brain response to social interaction represented by motion of simple geometric shapes in Heider-and-Simmel-type animations (Pavlova et al., 2010). The induced oscillatory MEG response of similar topography (localized to the left prefrontal cortex) peaked later in males than in females (Figure 1). These findings reflect sex differences in cortical processing of visually acquired social information. For females, anticipation of socially relevant events may be of higher biological, sociocultural and ecological value, and thus the left prefrontal cortex (a region known to be implicated in perceptual decision making) might be involved in expectation of the social environment and affective events by generating a template against which incoming sensory evidence is matched. In other words, females may anticipate social interaction predicting others' actions ahead of their occurrence, whereas males require accumulation of more sensory evidence before reaching social decisions. Without unveiling the time course of brain activity, it would be difficult to reveal these sex differences.

## Sex/gender, age and …and… and…

Research in healthy typically developing individuals most often overlooks possible (if not likely) sex/gender differences. Indeed, sex differences appear to be the norm rather than the exception. Usually, if not explicitly directed at investigation of sex/gender differences, both behavioral and brain imaging studies enroll a roughly equal number of female and male participants. Then the data are pooled together, and as a result we deal with the averaged “sexless” outcome that is then applied equally (poorly) to real females and males “in the wild.”

Without understanding the impact of sex/gender, age, social identity, and socio-cultural differences (e.g., Tanaka et al., 2010; Spencer et al., 2016; Hausmann, 2017; Koelkebeck et al., 2017; see also special issue on aging in *Frontiers Psychology: Frontiers in Emotion Science* entitled *Emotion and aging: evidence from brain and behavior*; Eds. Ebner and Fischer, 2014), it is impossible to make any progress in understanding affective behavior and underlying brain mechanisms in health and disease. The outcome of such research sheds light on complicated, contextually-dependent interactions of different major factors influencing affective behavior, and often counters popular wisdom and stereotypes! For example, females are widely believed to be more skilled in body language reading. Yet the pattern of experimental data in both typically developing healthy individuals and patients with deficient social abilities is “beyond simplicity” (Pavlova, 2017). Sex of observers affects body language reading in point-light movies depicting knocking at a door, but the effects are modulated by emotional content of actions. Women tend to surpass in recognition of angry knocking, whereas men excel in recognition accuracy of happy actions (Sokolov et al., 2011). At the same time, females exhibit an advantage in recognition accuracy of neutral actions that suggests that females are better tuned to the lack of emotional body language. A similar pattern of results was observed for subtle body language expressed by point-light human locomotion: The sex effects are modulated by the emotional content of locomotion and opposite actor gender (Krüger et al., 2013). Males surpass females in recognition accuracy and readiness to respond to expressions of happiness performed by female actors, whereas females show a tendency to be better in recognition of angry locomotion expressed by male actors. This may reflect biological and evolutionary significance of the opposite sex appearance with higher tuning of females to possible (even subtle) signals of threat or danger in body language of males, and higher tuning of males to happiness communicated through body motion of females.

The social brain impairments have many facets playing a key role in many neuropsychiatric conditions such as autistic spectrum disorders, schizophrenia, depression, eating disorders, and many others: all of them possess profound affective components. Most of these disorders are characterized by impairments in visual social cognition, nonverbal communication, body language reading, and facial assessment of social counterparts (e.g., Pavlova, 2012, 2017; Lazar et al., 2014; Pelphrey et al., 2014; Strauss et al., 2015; Pavlova et al., 2017; Yang et al., 2017). And many of these disorders (for example, autism spectrum disorders, Hull et al., 2017) also display a skewed sex ratio: females and males are affected differently in terms of clinical picture, prevalence, and severity. Currently, we are only beginning to understand the origins of sex/gender specificity of the most psychiatric and neurologic conditions.

## Author contributions

The author confirms being the sole contributor of this work and approved it for publication.

### Conflict of interest statement

The author declares that the research was conducted in the absence of any commercial or financial relationships that could be construed as a potential conflict of interest.
